# Pre-Exposure Prophylaxis Barriers, Facilitators and Unmet Need Among Rural People Who Inject Drugs: A Qualitative Examination of Syringe Service Program Client Perspectives

**DOI:** 10.3389/fpsyt.2022.905314

**Published:** 2022-05-30

**Authors:** Hilary L. Surratt, Hannah J. Yeager, Akosua Adu, Evelyn A. González, Elizabeth O. Nelson, Tamara Walker

**Affiliations:** ^1^Department of Behavioral Science, University of Kentucky, Lexington, KY, United States; ^2^Center on Drug and Alcohol Research, University of Kentucky, Lexington, KY, United States; ^3^Department of Anthropology, University of Rochester, Rochester, NY, United States

**Keywords:** HIV prevention, people who inject drugs, PrEP, stigma, rural, implementation science

## Abstract

**Background:**

People who inject drugs (PWID) are at high risk for HIV infection, yet in rural areas PWID are understudied with respect to prevention strategies. Kentucky is notable for heavy rural HIV burden and increasing rates of new HIV diagnoses attributable to injection drug use. Despite high need and the strong evidence for Pre-Exposure Prophylaxis (PrEP) as a gold-standard biomedical HIV prevention tool, scale up has been limited among PWID in Kentucky and elsewhere. This paper explores individual, environmental, and structural barriers and facilitators of PrEP care from the perspective of PWID in rural Kentucky.

**Methods:**

Data are drawn from an ongoing NIH-funded study designed to adapt and integrate a PrEP initiation intervention for high-risk PWID at point of care in two rural syringe service programs (SSPs) in southeastern Kentucky. As part of this initiative, a qualitative study guided by PRISM (Practical, Robust, Implementation, and Sustainability Model) was undertaken to gather SSP client perspectives on intervention needs related to PrEP, competing needs related to substance use disorder, as well as tangible supports for and barriers to PrEP uptake. Recruitment and interviews were conducted during September-November 2021 with 26 SSP clients, 13 from each of the two SSP sites. A semi-structured guide explored injection behaviors, SSP use, knowledge of PrEP, perceived barriers to PrEP, as well as aspects of the risk environment (e.g., housing instability, community stigma) that may impact PrEP uptake. Interviews were digitally recorded, transcribed verbatim and verified by project staff. A detailed coding scheme was developed and applied by independent coders using NVivo. Coded transcripts were synthesized to identify salient themes in the data using the principles of thematic analysis All study procedures were approved by the University IRB.

**Results:**

Participants were 96% white, 42% female, with a median age of 41 years (range 21–62); all reported injection use within the past month. Overall, we found low PrEP awareness among this sample, yet interest in PrEP was high, with several indicating PrEP is urgently needed. Clients reported overwhelmingly positive experiences at the SSPs, considering them trusted and safe locations to receive health services, and were enthusiastic about the integration of co-located PrEP services. Lack of basic HIV and PrEP knowledge and health literacy were in evidence, which contributed to common misperceptions about personal risk for HIV. Situational risks related to substance use disorder, particularly in the context of withdrawal symptoms and craving, often lead to heightened HIV injection and sexual risk behaviors. Stigma related to substance use and HIV arose as a concern for PrEP uptake, with several participants reflecting that privacy issues would impact their preferences for education, prescribing and monitoring of PrEP. Noted tangible barriers included inconsistent access to phone service and transportation. Primary supports included high levels of insurance coverage, consistent pharmacy access, and histories with successful medication management for other health conditions.

**Conclusions:**

Drawing on the critical perspectives of people with substance use disorder, our findings provide important and actionable information on individual and environmental barriers and facilitators of PrEP uptake among rural PWID at high risk for HIV infection. These data will drive the adaptation and implementation of a client-centered approach to integrated PrEP care within rurally located SSP settings to address unmet needs for PrEP care.

## Introduction

Despite decades of notable scientific advances in HIV prevention and treatment among highly affected populations (1) people who inject drugs (PWID) remain at high risk for HIV infection. A recent global review demonstrated that PWID continue to be severely impacted by HIV, with 9.0% of PWID in North America estimated to be living with HIV (2). Since 2015, HIV outbreaks among PWID in the US have occurred with increasing frequency in lower population rural communities (3–6), and Kentucky is notable for heavy rural HIV burden and increasing rates of new HIV diagnoses attributable to injection drug use (7). Nevertheless, rural PWID are generally understudied (8) and as such, critical information on uptake of HIV prevention services, including Pre-Exposure Prophylaxis (PrEP) is largely unavailable. Although numerous behavioral and structural interventions have successfully targeted PWID (9–14), virtually all have been in urban areas, and until recently most have not involved PrEP.

The World Health Organization added PrEP to the recommended combination HIV prevention package for PWID in 2014 (15) and in 2015 issued guidance for PrEP implementation in PWID (16). To date, the strong scientific evidence-base for PrEP as a gold-standard biomedical HIV prevention tool has not translated to optimal clinical care, with scale up in the US modest overall (17), and particularly among PWID (18). Kentucky is no exception, with an estimated 11.4% of individuals with an indication for PrEP receiving PrEP coverage in 2020, one of the lowest rates in the nation (19). Barriers to PrEP implementation among PWID are multi-level. At the individual level, awareness of PrEP and perceived risk for HIV is modest in recent studies with PWID (20, 21). Noted structural barriers include the cost of obtaining PrEP medications, housing instability, and lack of secure medication storage options (21–23). In rural areas specifically, structural barriers include long distances to PrEP providers, limited availability of health care providers and testing sites in general, and high levels of stigma surrounding HIV factors (24–29). Clinical barriers in rural healthcare sites also reflect poor infrastructure and capacity for PrEP delivery, lack of PrEP knowledge among staff, and absence of local PrEP providers (30) leading to PrEP “deserts” (31, 32). Although supports for PrEP uptake among PWID are less widely described, Allen et al. (21) recently found that integration of PrEP services into venues that PWID routinely access would help to optimize PrEP awareness in communities where there is low background knowledge of PrEP.

This paper explores individual, environmental, and structural barriers and facilitators of PrEP care from the perspective of PWID in rural Kentucky. Using qualitative approaches guided by the PRISM (Practical, Robust, Implementation, and Sustainability Model) implementation science framework (33), we elicited PWID's perspectives on their risk environment (34–36), as well as sources of support and preferences for PrEP care access that may influence PrEP uptake. Rural Appalachian PWID are situated in environments characterized by high levels of stigma related to substance use (37), unstable housing, fear of arrest, economic distress, and inadequate access to services (38–41), which underscores the need for interventions that address multi-level barriers to improve HIV prevention outcomes. Nevertheless, rural PWID also demonstrate notable resilience and motivation for health improvement, including uptake and consistent use of SSPs to obtain sterile injection equipment (40). This manuscript examines rural PWID's lived experiences to systematically assess PrEP barriers, facilitators and unmet needs, which will inform and guide adaptation of a PrEP-focused intervention to expand access in rural care settings.

## Methods

Data are drawn from an ongoing NIH-funded implementation study designed to adapt and integrate a PrEP initiation intervention for high-risk PWID at point of care in two rural Appalachian syringe service programs (SSPs) in southeastern Kentucky. We used the PRISM (Practical, Robust, Implementation, and Sustainability Model) framework (33) to guide this project. PRISM assesses organizational and individual level contextual factors that may contribute to implementation outcomes, specifically examining elements of the external environment, program or intervention design, implementation and sustainability infrastructure, and the multi-level recipients of an intervention (organizations, providers, and clients) to understand barriers and facilitators to implementation.

### Study Sample

Participants were recruited through in-person contacts by the study team who were present on site during operating hours of the participating SSPs. These programs are integrated into regular health department operations in Knox and Clay Counties in rural southeastern Kentucky; the SSPs have been operational since 2016 and 2017, respectively. In addition, the Clay County Health Department operates a mobile SSP 1 day per week in a remote location to provide sterile syringe access in outlying areas. Both Knox and Clay Counties are entirely non-metropolitan based on Rural-Urban Continuum Code indicators. Eligible participants were age 18 or over and reported use of the SSP and injection drug use at least once in the past 30 days. Twenty-six PWID participants enrolled and completed qualitative interviews between September and November 2021.

### Study Procedures

Study enrollment and in-depth interviews were conducted in two county health department fixed site SSPs, as well as one mobile site. Brief study eligibility screening was conducted by a study team member, which included collecting age and other basic demographic information, as well as questions on recent substance use patterns and SSP utilization. Study staff reviewed informed consent materials and discussed the provisions of the consent document prior to beginning the interview. Participants were asked to provide written consent that they agreed to participate in the interview and agreed to audio recording.

An experienced qualitative researcher facilitated the one-on-one interviews in a private room within the fixed SSP locations, and at a private outdoor space adjacent to the mobile site. These semi-structured, in-depth interviews were organized by an interview guide, focused on the PRISM domains of *clients as recipients of the intervention and client perspectives on the intervention*. Key topical areas included: injection behaviors, HIV risk, SSP use, knowledge of PrEP, perceived barriers to PrEP, physical and mental health care access, use of HIV prevention services, social supports, strengths and resilience, as well as aspects of the risk environment (e.g., housing instability, community stigma) as they impact PrEP uptake. Questions related to PrEP awareness and interest were asked in the final segment of the interview, and were introduced with the following item utilized in prior research (42): *Have you ever heard of HIV-negative people taking a pill every day to reduce their chances of getting HIV infection (this is called PrEP, for Pre-Exposure Prophylaxis)*? For clarity, participants were simultaneously shown the *PrEP 101* consumer fact sheet developed by the Centers for Disease Control and Prevention. Interviews lasted between 30 and 60 min. A $30 gift card incentive was provided to participants upon interview completion. Institutional Review Board approval for the study was obtained from the University of Kentucky Medical IRB.

### In-depth Interview Data Analysis

Four primary steps were taken to analyze the textual data elicited in the in-depth interviews. These included: (1) initial verbatim transcription and verification of interview audio recordings; (2) focused readings of these transcripts; (3) the construction and application of a detailed coding scheme; and (4) the compilation of core explanatory categories from the analysis of the transcripts and the construction of an interpretive summary based on the interview codes. Interviews were recorded and transcribed verbatim using a HIPAA-compliant transcription service. Interview transcripts were then reviewed and verified for accuracy by a member of the research team. Guided by PRISM domains, and an initial reading of the transcripts, the research team developed a coding scheme for the interview data in NVivo (43), using a hybrid deductive-inductive approach (44). Initial codes were primarily deductive, sourced largely from the relevant domains of the PRISM framework and initial readings of a small subset of transcripts. Subsequently, inductive approaches that drew on salient information in the raw data were utilized to further develop the codebook and account for new or unanticipated patterns of responses. Several members of the study team are experienced qualitative researchers and served as independent coders; at least two members of the research team coded each interview transcript. The research team met weekly to discuss coding progress and achieve consensus on coding consistency, and to evaluate whether new codes were identified that indicated novel emerging themes, and whether existing codes needed further refinement. The coded transcripts were merged and synthesized to identify the primary themes in the data using the principles of thematic analysis (45).

## Results

Table 1 displays basic demographic, health, and social characteristics of the interview participants. Overall, participants were 96% white, 42% female, with a median age of 41 years (range 21–62). All reported injection use and SSP use within the month prior to interview, including one participant using the SSP for the first time on the day of interview. Twenty-one participants (80.8%) reported using the SSP for at least 1 year, and 19 (73.1%) reported visiting the SSP at least monthly. Nearly three quarters of participants reported histories of formal substance use treatment, involving either residential or outpatient care, or medication treatment involving buprenorphine. Additionally, more than half described experiences involving arrest, detention, and incarceration, indicating significant histories of contact with the justice system among the individuals interviewed. Notably, nearly 60% reported Hepatitis C (HCV) positive status tied to risky injection practices. Approximately 80% of participants had ever been tested for HIV, and the majority (61.5%) had done so within the past 6 months at the SSP. Overall, there was low baseline PrEP awareness among this sample (26.9%), yet interest in PrEP was high, with 73% indicating a desire to learn more about PrEP for personal use.

**Table 1:** Syringe service program participant characteristics and pre-exposure prophylaxis perceptions (*N* = 26).

	***N* (%)**
Demographics
Gender
Female	11 (42.3%)
Male	15 (57.7%)
Race/ethnicity
Black/African American	1 (3.8%)
White, non-hispanic	25 (96.2%)
Age (*Median; Range)*	41 (21–62)
Current drug injection*
Methamphetamine	9 (34.6%)
Buprenorphine	15 (57.7%)
Heroin	3 (11.5%)
Prescription opioids	1 (3.8%)
Health and social factors
Current health insurance	26 (100%)
Arrest/incarceration history	14 (53.8%)
Hepatitis C positive	15 (57.7%)
Healthcare services utilization
Lifetime substance use treatment	19 (73.1%)
Currently prescribed buprenorphine	8 (30.8%)
Lifetime HIV testing	21 (80.8%)
PrEP perceptions
PrEP awareness	7 (26.9%)
PrEP interest	19 (73.1%)

*Adds to >100%, responses not mutually exclusive.

### Barriers and Supports for PrEP

Our systematic examination of barriers and facilitators of PrEP care revealed several key themes related to uptake of this HIV prevention tool. Table 2 displays a summary of the primary themes that emerged in analysis mapped to the relevant PRISM domains that were activated.

**Table 2:** Primary Themes for HIV Prevention and PrEP Uptake among Rural PWID Mapped to PRISM Domains.

**PRISM domain**	**PRISM element activated**	**Theme**	**Example quote**
Recipients, client characteristics	Knowledge and beliefs	Need for HIV information	*There ain't no AIDS around here. If there is and they're made aware of that*.
		Uncertain personal risk	*I get scared thinking if I got it, I don't know if I want to find out*.
	Disease burden	Substance use disorder situational risks	*When you are sick and withdrawing there's no line you won't cross*.
Program (intervention), client perspectives	Addresses client barriers	Stigma: social rejection and harms from systems	*The stigma would be like that. You wouldn't want somebody to know you're taking it [PrEP] because they think you have HIV*.
		Scarce physical capital: pervasive economic distress	*Sometimes I might miss a week cause I ain't got, my ride got tore up and you know, you might not find a ride up here*.
	Access	SSP utilization: safety, inclusion supports expanded care opportunities	*I felt like that I was being taken care of, that someone cared enough that I didn't have to shoot with used needles*.
	Client-centered	Health management: resilience, empowerment, readiness	*I had it [HCV]. And then I took the Mavyret and got rid of it. I took three pills a day for two months*.

#### Barriers: Knowledge and Beliefs

Overall, a lack of basic HIV and PrEP knowledge was in evidence among interview participants. Participants were uniform in stating that they did not have exposure to HIV prevention education or messages in their communities of residence and did not hear HIV discussed as a priority health issue. In fact, even among those reporting awareness of PrEP, their exposure was often incidental through media, advertisements, or other sources outside of their home communities. An emergent theme in participants' narratives centered on an *unmet need for HIV-related information, and related uncertainty in gauging personal risk and managing prevention*. In this regard, participants largely relied on informal or sporadic sources for HIV prevention information that framed their views and concerns around risk. This uncertainty was consistently noted as a source for complacency and ambivalence by several participants:

*I don't know, you don't really hear much about it. You just hear people talk about people. But you know, you don't hear much about it. You really don't. A lot of people don't think about it. (Male, 50s)*

*There ain't no AIDS around here. If there is and they're made aware of that. That would make this that much more important to them. Uh, most people, when you hear AIDS you think of homosexuality, you think of cities, you don't think that the good old boy out in Gertler, Barbourville has it. You know what I mean? Uh, I think maybe if they knew how prevalent it was, or even if it, I don't know if it's prevalent around here even. And I pride myself on being informed. (Male, 40s)*

This context of uncertainty fueled by limited information pervaded personal risk evaluations as well. Many participants expressed fear of HIV due to the lack of a cure, uncertainty about testing and treatments, and relied on informal awareness of HIV-positive individuals in their small communities to understand prevalence:

*I used to, and I get scared thinking if I got it, I don't know if I want to find out. But now you're more likely to die with it, than from it. You know what I mean? A lot of people still see it a death sentence, I guess. (Female, 40s)*

*But I actually probably need to do it again. It's been a long time. I don't feel, I feel all right. For some reason, I can't gain no weight. I eat a lot. And I'm not gaining weight. I don't know why. I don't think… I think I don't have it, but I shouldn't think like that. I need to get tested, now that you say that. I mean, I want to. Just to see, to make sure I don't have anything. (Male, 50s)*

*I get nervous every time, no matter what, getting tested for stuff like that. That's the most terrifying thing. Like back in the day I watched people die from Hep-C. So when I got it, I flipped out and now they have a cure. I mean, but HIV they're really behind. Not behind, they're catching up really well with the treatment. Now there's treatments they can live with it, right? (Female, 30s)*

*Yeah, I've got a couple friends with it. Yeah, I got a girl, and her man, and another one, and another one, and another one. Yeah. I know five or six got it. I walked up to their house and I was going to smoke a joint with them. And they said, “Hey, probably best you just give me a joint.” I said, “Why?” They said, “Because I got Hep-C and HIV.” I said, “Well, how'd you catch it?” They said both sharing needles with each other. (Male, 30s)*

With some exceptions, participants were largely cognizant of both sexual and injection-related risks for HIV but most perceived their personal risk as modest, and markedly lower than in the past. This shift was largely attributed to uptake of the syringe service programs, noted as structural facilitators of reduced injection related risks and sharing behaviors:

*I lived in a trap house and there was a hole in the wall and that's where we put our rigs and you just reached in and got one. Uh, and at the time, you know, I was wanting to die anyway, and I really didn't care. So if there had been an exchange over there, I know it would've made a difference. I know it would have made a difference. Maybe not necessarily to me specifically, but at least one person. It would have saved one person from having Hep or HIV. (Female, 30s)*

Nevertheless, several participants were candid about episodes of ongoing injection risk that remain, identifying aspects of *substance use disorder severity as critical to unanticipated situational risks*. Situational risks were most apparent in the context of withdrawal symptoms and craving, which were often tied to heightened injection risk behaviors:

*I remember me not being able to get my shot and I was sick for like four days. And subutex, suboxone withdrawals, that's a whole other story, it hurts, it hurts your bones. Um, but I remember I didn't get up off the couch. I was trying to go [inject] in my hands, and I didn't get up to do, to rinse it out or nothing. I just kept on trying and trying and trying. (Female, 30s)*

*When you are sick and withdrawing there's no line you won't cross. (Male, 40s)*

*Yeah, it's such an overwhelming urge when you're sick, you feel it, uh, I could explain it a hundred different ways and, and I hope to God, you never have to experience it with yourself or any of your loved ones. There's no stronger of a driving force than a detox, than a withdrawal. (Male, 40s)*

#### Barriers: Stigma and Rejection

The background experience of interview participants as PWID, members of a highly stigmatized group within small rural communities, was apparent in many aspects of their narratives. A key theme in this regard related to *social rejection from both individuals and systems, and lived experience of harms from systems*, be they justice systems, treatment systems, or healthcare systems. Interview narratives reflected a deeply felt absence of community membership, or social capital, with several participants describing their location in marginal spaces on the boundaries of the community. Individual accounts of justice issues are illustrative of systems harms that shape individuals' experiences of safety and surveillance:

*We can't get no help from the police. Because they hate on us because around here, if you don't come to ‘em with your hat in your hand. (Male, 50s)*

*I'm harassed on a daily basis here. I mean, when I pull out of a gas station down in town, they all just turn their lights on. I about wrecked the other day, and they pulled my britches down in public trying, looking for drugs at a mother fucking gas station. I'm allowed. I mean, that's not legal. And they're a bunch of kids, that's what it is. But somebody's telling them what to do. And because they don't even know me. (Male, 50s)*

*They used to harass me a lot but they don't no more. Me, my family before that. It's just, I don't know. They just didn't like our last name or something. I don't know. Which, you know, I did get pissed at ‘em because they lied on me and they tried to send me to prison and stuff for stuff I didn't do, and it really made me angry. I hate ‘em for it. Like, uh, if somebody shot me up there, we wouldn't call the cops. I just don't like ‘em and they don't like me. The reason I don't like ‘em is because you always got the pricks you got to deal with. They want to judge you and they want to accuse you of stuff you ain't done. And so I don't fool with them and they don't say anything to me no more. I have no trust with them whatsoever. They'll figure out something to charge you with. I guess it felt like that all my life. The issues is if you're not kin to them or a snitch, they don't like you. (Male, 40s)*

*They arrested me on that possession and the paraphernalia charge. They still go stack 29s [warrant checks] on me at least three times a week. To see if I got warrants on, and I've been out of jail two years or longer. And they still three, four times a week. Still to these days they'll run my name, every time they see me. They stop me. (Male, 40s)*

These pervasive stigmatizing interactions have important implications for understanding uptake of treatment and healthcare among PWID, including PrEP. Adverse experiences in treatment settings were commonly reported, with interview narratives describing these episodes as inappropriate, unhelpful, or even directly harmful, creating feelings of mistrust, humiliation, and injury. Interview participants noted deficits in accessing care that was evidence-based, that allowed medication, or that followed best practices for retention in care, which frequently resulted in internalized stigma:

*I felt deceived at the place that I went to. I graduated. I did everything that's asked of. And they asked me, said, what was you going to do when you graduate? I said, I'm going back home to my wife. And they said, well, we don't think that's a good idea. I said, well, you asked me what I was going to do. Well then, we think you should take the second program here. I said, I want to talk to my PO and I said, Hey, do I have to? Because prison is the one that sent me. And he said, no, you just have to go through phase one. I said, do they know that? He said, yeah, they know that, but they try to make you think. And he was just honest with me. (Male, 40s)*

*Yeah, they have 100 people. If somebody messes up, they don't get to go outside and smoke or nothing. So, you have to get up at five o'clock in the morning and make your bed, and I never did like orders, so I didn't get along with it. They get up and you got to tell them what choice of drugs you got to do, in front of 100 people. And I was the type that, hey, I'm a drug head. I mean, it's simple. I mean, I don't want to talk. And I didn't want to look stupid in front of 100 people, because here I am in rehab for the same reason. Drugs. And yeah, I got in there and I stayed, like, 15 days out 90, and got kicked out. They kicked me out. They said I had suboxone in my system. And they wouldn't give me my Seroquel, so I had to come back to Clay County, like 200 miles away and get my Seroquel. They didn't know the pharmacy number, they didn't know what they was doing. So, yeah, pretty much they was trying to make a joke out of me. I'm afraid to go back to a suboxone clinic, where I'm court ordered, they'd probably put me in jail. So, I'm scared. (Male, 30s)*

*The courts kind of screwed me. They said all I had to do was complete a month and then they'd take my felony off. Well, I completed the month, easy, and the people said, “Well, you're doing so well. Uh, how would you like it if you stayed for the 90-day program?” So, I voluntarily stayed for 90 days, but they ended up switching my stuff without telling me, “Well you was only court ordered for 30, but since you want to do 90, we're going to court order it for 90”. And then I ended up taking off and I kept my felony and lost my marine corps chance. (Male, 20s)*

In a similar way, many participants experienced rejection or exclusion in a variety of healthcare settings that led to subsequent avoidance of care, unwillingness to seek help for acute and chronic health problems, and inability to effectively engage in or uptake disease prevention activities.

*Yeah, people, I know people who had abscesses on their arms. They don't go to the hospital cause they talk about ‘em. (Female, 30s)*

*If you ever have to go to the hospital here for something serious, and you go there for something. And they say, well, you got this in your system. Well, yeah, if you would've asked me that, I would've told you. You didn't have to try to trick me into do anything, but I'm here because of this, not because of that. And then they look at you totally different….Can you fix what's wrong with me? That's all I want. So I said, first of all, before I even pee in cup, I smoke pot. I do get high, but I still need help for this. Can you guys help me? (Male, 40s)*

*I met a doctor two, three days ago that very badly upset me. It was a gynecologist and she said, “When was the last time you shot meth?” I said, “Excuse me?” She said, “When was the last time you shot up meth?” I said, “I don't do meth.” I mean, that was very upsetting and I didn't think she was even allowed to ask me something like that. This is the first time I've ever met her. So actually I don't want to meet her again. She made me feel like I was about “…that big.” (hand gesture indicating small size). (Female, 50s)*

*At the emergency room they will barely even give me an ibuprofen because where I inject drugs and stuff like that. And one time I sprained my ankle, well that's why I'm walking around with a sprained ankle right now because, uh, they ain't no sense in going over there cause they ain't gonna do nothing for me. Because where I've been injecting drugs, they got it wrote down in the paperwork that I'm an IV drug user. So they won't give me nothing for pain and nothing to help me out. So I have to get mine off the street. (Male, 30s)*

*You go to a pharmacy here and you ask to purchase them [syringes]. Right. They're going to shut you down real quick. They don't want to help you. (Female, 30s)*

Background experiences of stigma and social exclusion had a strong connection to HIV prevention attitudes among interview participants. For some, HIV was feared as another potential source of social rejection, while for others, HIV prevention methods were also seen as potentially stigmatizing, by indirectly disclosing involvement in injection or sexual risk behaviors. Stigma related to HIV arose as a direct concern for PrEP uptake, with several participants reflecting that privacy issues would impact their preferences for education, prescribing and monitoring of PrEP. For some, stigmatizing experiences with HCV diagnosis were called to mind, driving the prioritization of privacy around PrEP care for HIV:

*They shun people with it. You know, I don't judge no one. Who am I to judge? I mean, I may not like what you're doing, but I don't dislike you for it. (Male, 50s)*

*Oh, if someone has it, they'd probably talk about them like a dog. It'd make it, it'd be a hard time on ‘em, they'd probably have to move away. To be honest with you, they look at you different, down on you, you know. (Male, 50s)*

*It would be terrible because I was just about the first person in our little holler that tested positive for Hep and I got treated awful. I mean, people, family ignored me, old friends ignored me because they were scared to death. I mean, they didn't know what it was so, which I was scared to death too. (Female, 50s)*

*I'm sure they wouldn't share it. You know what I mean? That's a very private, like, even me having Hep-C, I would never ever tell anybody, like, I'm very ashamed of it. I'm very embarrassed. So I'm sure it's the same with HIV. You wouldn't want to be around ‘em. I mean, when like you're sharing needles and like, “are you sure you don't have HIV”? I got Hep-C, that's cool. I mean, that's how crazy it is. (Female, 30s)*

*The stigma would be like that. You wouldn't want somebody to know you're taking it [PrEP] because they think you have HIV. (Female, 30s)*

*If it's something to do with you having it [HIV], you know, then you might want to keep quiet. But no, this is telling them, you know, you're trying to just prevent it, I mean. I don't see nothing bad there. (Male, 50s)*

*I don't know. I'd be kind of embarrassed just to go get it [PrEP]…people just love to talk in general. (Female, 50s)*

#### Barriers: Scarce Physical Capital

Participants commonly mentioned experiencing strained and scarce personal financial resources, tied to the broader community landscape of declining economic prospects. Even among participants who were not personally impacted by severe economic hardship, interview narratives reflected a palpable theme of *pervasive economic distress and poverty as drivers of risk* for individuals in these rural communities:

*I don't know why this area, so there's nothing here. I get that. There's no, there's no, uh, way to prosper, or like have, any kind of future here at all. And I get that, but why has it been so long that it's been going on? This is not a bad place to live. As far as the area is now. It's very pretty here, but there's just not enough. I feel like there's something that holds this area back and I just don't, I'm not sure exactly what it is. Now it's the drugs and you know, people in the, where, where did it all come from? That had to start somewhere because it didn't just happen overnight. It happened over a long period of time. Why didn't we move along like everybody else in the country? (Female, 30s)*

*I think all these people around here that if you ain't rich, then you can't survive. It's rough*.

*Like you get pretty much, most people that ain't got money or jobs or stuff like that, they end up homeless, on the streets, and then they end up in jail because they got homeless on the streets doing drugs. (Male, 30s)*

While housing opportunities were most often available due to the presence of supportive family members and extended family networks, many participants reported scarce financial resources, income, and employment opportunities that inhibited consistent access to cell phones and personal transportation, which are especially critical in areas that are largely devoid of public transportation systems. These factors were reported as the most common tangible barriers to communication and attendance at health-related visits:

*Yeah. I'm kind of working on getting a phone. That's been my biggest thing about the doctor and everything. Because I'm having to…I mean some family members in the house could do, but it's hard to get to use their phone. (Male, 40s)*

*She was going to call me, and my boyfriend got my phone. I have the worst trouble with him with my phone because he will not leave my phone alone. He thinks he has to have it and use it. It's like we go through a phone a month, me and him do sometimes. (Female, 50s)*

*I'm still trying to. I might have one today maybe. I actually, I can get a phone up at Walmart for, I think $30 is the lowest one you get up there. I think about going straight up there and just getting a phone. So that would take a lot of stress off of me because at the doctor would have a number that they call. (Male, 40s)*

*Sometimes I might miss a week cause I ain't got, my ride got tore up and you know, you might not find a ride up here. (Male, 30s)*

*Just no transportation. I mean, getting there. (Female, 40s)*

Although not highly prevalent in this sample, for individuals reporting unstable housing it represented a highly salient barrier to healthcare and PrEP services uptake, deeply impacting all aspects of personal ability to connect with services, to follow a medication regimen and safely store medicines:

*Well, I'm homeless. I'm homeless, I'm living in a tent. I have to wonder from day to day where I'm going to be able to lay down, how I'm gonna feed myself, uh, this or that. And how I'm gonna feed my drug at that. Uh, uh, and it's just, a million things going on in my mind everyday. And then I move my tent, like every other day, probably. Cause if you don't, somebody will take it and it's sad to say, but people will take the place you are now. So you have nothing when you come back. (Female, 30s)*

#### Supports: Syringe Service Program Utilization

As noted earlier, interview participants were recruited from the local syringe service programs in their counties and were current participants in the SSPs. Participants were both novice and experienced SSP utilizers, but most had used the programs for at least 1 year. Participants reported overwhelmingly positive experiences at the SSPs, considering them trusted and safe locations to receive health services. It was quite common to report initial hesitation about the programs due to privacy concerns and concerns around law enforcement activity, but these diminished over time:

*I was kind of worried about the cops. That's what scared me. That's what really made me nervous was going to jail. (Female, 50s)*

*I believe that the policeman used to sit up across the mountain over there and watch and see who came here, when it first started doing it. And I don't think that's right. (Male, 50s)*

*I wasn't too sure. I don't know, I really never thought much about it. I kind of thought the law or something watched or stuff like that. Because the stuff we do ain't legal, but I was a little concerned always watched for the law and stuff. (Male, 50s)*

*Well, they were in the back place a lot of times you'd see an unmarked car or a cop used to sit back there. There is one that worked there or something. We didn't know and then you've got people that talk stuff like “they follow you home”. And then, you know, sometimes you start wondering because you don't do that stuff that you should do, like selling, stuff like that. So, then you leave here and you don't know what's going on. (Male, 50s)*

*The biggest difficulties coming to exchange was the fear of people finding out. (Male, 50s)*

Key in the trust building process were assurances from peers and program staff about the confidential nature of the programs. Several participants reported being initially referred to the programs by friends and now trying to encourage others to utilize the programs or exchanging for others who are still reluctant to attend. They valued the confidentiality protections of the programs, and privacy was once again highly valued in these small communities. Several clients mentioned being fearful of disclosures if using the program, but none had experienced this.

*When people don't understand anything, they pretty much talk negative about it. It goes along with everything, and I've heard a lot of people say I wouldn't go down there for nothing. And I said, well, if you got old ones, give them to me, I'll go up there and I'll exchange it for you. And when I come up here, I tell the ladies that give them to me, what I do with them. I say, when I go up there, I don't use it that many a day, but I help other people out. (Male, 40s)*

*The lady that was here kind of was like… She didn't come right out and say, “Well, no, we're not going to call the cops or anything like that and tell on you for changing needles, but you do need to have clean needles. That's where we're trying to help you, with trying to get you clean needles and keep clean needles, keep you in the program as long as you do drugs to where you will have clean needles. You won't be injecting with dirty needles and stuff.” (Male, 40s)*

*Well, they don't take your name. Well, they just use the first two letters of your first name and last name. (Male, 30s)*

*My friend. He come up here and showed me the ropes around here. He introduced me. He's family, well, he ain't family, but he's like family. (Male, 30s)*

*I started coming at the beginning, let's see here, about two years ago. I found out about it because I was buying needles off people. And then I just got to where I was like, well, if I don't go over to the exchange, and I keep doing what, I'm going to be right back in the hospital for the same crap, you know what I'm saying? I don't want to go through that again. They're always nice to me. And I was worried because I know some of the people, I live around some of the people. I was afraid they might say something to my mom, you know what I'm saying? But they haven't, thank God. Because she would kill me. (Female, 30s)*

Participants noted many important benefits of using the SSPs, not surprisingly, enhanced access to sterile needles was consistently reported as the primary benefit of these programs. In these small communities, SSPs were often noted as the only source for obtaining sterile injection equipment:

*You was having to buy these needles and stuff and go to people, they're going to charge you a dollar a piece for them. Some people charge $2 a piece for them. You have to end up paying for them every time you get them, and sometimes they won't have them and stuff. I'm afraid they'll give me a dirty needle and say it's clean or something. It kind of worried me if I ever had to. (Female, 50s)*

*Well, to be honest with you just trying to get needles because it's hard. You can't just go to Walmart here or any place to get them. So you start buying them off people around here and Lord, you pay five, six dollars a needle. So, I did start reusing needles. And so, uh, just from here, they helped out with everything. They really helped out with everybody*. *(Male, 50s)*

*I did [shared] when I first started. It's been years ago now. Now I just use a brand new one, one time and when I'm done, I store it and take it to the exchange. And there weren't no blood or anything in it. But I would put bleach in it, clean it out in water and then take a lighter, heat the end of it, that like, it takes all the skin cells and stuff off of it and then do it… we just couldn't get new ones [needles]. (Male, 20s)*

*I didn't actually share needles. I shared a can that we made enough for three shots, and we all pulled up out of it. You know what I mean? I really don't think I've shared. I've never shared a needle with anyone, but I've used my own needles. Because back in the 2000s, you couldn't get needles like you get them today. And that's why my arm is scarred up a lot too, because they're so dull. And I mean, I've used dull needles. So now, I mean, I'm not proud of it, but it is what it is. (Male, 50s)*

In a general way participants described the SSPs as overcoming a long-standing structural risk related to lack of access, and for some this assurance represented an opportunity to be intentional about other health changes, including testing and treatment. Many participants mentioned that using the SSP had allowed them to reduce sharing and re-use behaviors, which they credited with prevention of HCV and HIV, but also acute illnesses and infections:

*I've not caught any new diseases and I'm not having to reuse and reuse and cause all them sores on me from, you know, reusing. (Male, 30s)*

*It [HIV] was on my mind a lot and, uh, um, I guess, um, me being crazy or whatever. Um, never thought about, you know, going to the doctor or something and just went in and start asking for an HIV test. But that's been a real help, like to put my mind at ease. So it's a little off my mind since then, since they started to do it [testing]. (Female, 30s)*

*I wouldn't have otherwise known, like I know I could go to the doctor or whatever, you can go to the hospital and walk in and say I want to be tested, but other than that, generally I wouldn't off the top of my head think of where to go. So that's a good thing that they have. I wouldn't, I wouldn't have had it done. I wouldn't have had it done any time otherwise. (Female, 30s)*

*When I started the program [SSP] I thought, well, you know, I'll always have new needles. So I went and got Mavyret [HCV treatment]. (Male, 30s)*

Interview narratives related to SSP use crystallized an underlying theme regarding *enhanced safety, inclusion, and community that supported continued SSP use, but also integration of PrEP care*. Participants conveyed a sense of safety, belonging and dignity that pervaded interactions at the SSP and supported meaningful engagement in care. Issuing of SSP cards by the health department was also seen as providing protection from law enforcement, empowering participants to safely and legitimately possess injection equipment:

*I like all the people here, they're my friends outside, so I know everybody. (Male, 50s)*

*I felt like that I was being taken care of, that someone cared enough that I didn't have to shoot with used needles, bad needles. (Female, 50s)*

*Everyone over there is fantastic because they're all very personable. They're, uh, very knowledgeable. They work with you, you know, they're not like dismissive, you know, they're just a terrific bunch and things, what I've seen. (Male, 50s)*

*He worked here for a long time. He was good person ‘cause he been through the same thing and got saved. And he knew what we're going through. I mean, you know, they don't look down on you. So I always liked him. That's what got me coming here. I was comfortable with him and all of the ladies working here were good. And you know, some places people look at you like a piece of trash or something. Look at you like you're different, but they're always good to me. (Male, 50s)*

*I've been stopped and had syringes, needles on me. As long as you tell them that you got the needles and where they're at, they'll put them in a container and not charge you with them or whatever. They give us a card, but I can never keep up with it either. They give us a little break*. (*Female, 40s*)

These positive care experiences at the SSP set the stage for receptivity to integrated PrEP care among participants. As noted earlier, interest in PrEP was high, with 73% indicating a desire to learn more about PrEP for personal use:

*Hell yeah. I mean, if that'd keep me from catching anything, yeah. Hell yeah. Because I had my shots whenever I was little. I might take you up on that pill. For real. (Male, 30s)*

*I would [be interested]. I would get some people together and try to bring them up here*. I know at least two people would come with me. There'd be three. (Male, 40s)

*I'd like to take it. How could you get it? I would rather do it without going to my doctor, to be honest with you…it would be great if you could do it here. I am very interested in it, and I know a bunch of people that would be interested in it. If they could do that with the program, the needle exchange, and just offer it. (Female, 30s)*

#### Supports: Successful Health Management

Participants reported a high prevalence of health complications, including HCV infection, overdose, abscess, sepsis, endocarditis, and chronic diabetes, lung, and liver problems, indicating significant life challenges related to co-morbidities. Although these health conditions clearly represent serious stressors, we noted an emergent theme in the interview narratives regarding these background experiences of illness, which particularly with other bloodborne infections, raised awareness of vulnerability and highlighted the value of prevention. Participants expressed *resilience and feelings of empowerment in successfully managing existing health conditions*. Several described taking regular medications for health issues, which supported their readiness and agency to manage PrEP:

*I had it [HCV]. And then I took the Mavyret and got rid of it. Three months. I took three pills a day for two months. They said it might give you a headache or something, but I didn't have no side effects. You, but you had to take it every day. If you missed a day, it won't work, well they said it wouldn't but it did. It's wild, it did. (Male, 30s)*

*I have chronic Hepatitis C. I did have, and I took the medicine to clear that up again. I think I've contracted it back, maybe. Uh, I was tested for HIV just today, but then it was negative. And then, uh, the last time I was tested for Hepatitis C it was over here at the hospital. It was about a year ago. I cleaned it out, cleared it out, took the medicine. (Male, 30s)*

*With high blood pressure, cholesterol and things like that in general, you know, that is an ongoing thing. As far as conditions, you know, you'd have to have medication sometimes for the rest of your life. So yeah, it is, you know, a continuous process and everything. I'm diligent about things like that. You know, the doctor says take it, you know, because that way I don't go off on a different path. (Male, 50s)*

*I have high blood pressure. I get, that's why I get high blood pressure medicine. I take it every evening. I've been getting it for about 3 years now. (Male, 30s)*

*I had got an infection in my heart, stayed in UK [University of Kentucky] for seven months. It shut my kidneys down. I'd done a bad shot. I had to learn to re-walk. But I got kicked out because my husband come up there, out of his mind, and I didn't get to do my last treatment. So when I come home, I still was septic. Then I got out and had to go back to Pikeville and do heart surgery. And it's the whole backside of my heart, or valve or something. My heart rate's fast. In fact, my whole life. And when they done the heart surgery, it makes my heart overwork. So I take two or three different blood pressure pills, but I ain't got a blood pressure problem. My mother makes sure. I got three girls too. Trust me, they all make sure I take them. (Female, 30s)*

Successful health management experiences were often tied to more robust levels of social and physical capital that promoted access to care, insurance, and pharmacy benefits. Critically important social support from relatives or other trusted persons arose as valuable for health promotion as well. In the context of stigma related to substance use, injecting behaviors and HIV risk, participants often expressed a tension between privacy and disclosure, generally preferring to keep their behaviors personal but also selectively seeking safe spaces for disclosing issues around substance use. For participants who had a provider or family member with whom they could openly disclose their substance use and health concerns, there were tangible benefits that optimized their healthcare that may also support PrEP care:

*I go to them monthly. I have high blood pressure. I get, that's why I get high blood pressure medicine. She's a good doctor, yeah, they treat you well. Well, I sort of, I know her too. I grew up with her kids. I know who she is, so I did feel comfortable with her. (Male, 30s)*

*Um, um, my, well, my doctor is, um, um, one of my good friends. I've been friends with her pretty much all my life. She lived next to me when I was seven or eight. Now she's a nurse practitioner. So she knows what I do. (Female, 30s)*

*I have an older sister that lives here in the community and things, and she's very supportive because she's been in the healthcare profession. So if I was to have issues, as far as getting somewhere transportation or anything like that, you know, well, I'm there for you, she'll take me and, you know, like pick up my medication or anything like that. (Male, 50s)*

## Discussion

This study employed qualitative methodologies guided by PRISM to identify the salient personal, social, environmental, and structural barriers and supports for PrEP uptake among rural PWID, with the goal of informing PrEP intervention efforts tailored for this population. Interview narratives with PWID attending rurally located SSPs captured the lived experiences and engagement of individuals in these programs, and in many cases documented long-standing histories of addiction, significant burdens of substance use disorder, multiple health complications, scarce economic opportunities, and loss of community due to multi-layered experiences of stigma and discrimination. Despite these challenges, however, participants also expressed significant resilience and strength, intentionality, and motivation to engage in HIV prevention.

We found a pervasive gap in locally available HIV information; for all intents and purposes messaging about risk, transmission and prevention was very limited in these rural communities. Consequently, perceptions of risk were generally modest and PrEP awareness was minimal among the PWID we interviewed. We did not find systematic differences in HIV knowledge or risk perception by participant age or gender. Given this very limited exposure to the topic, it is not surprising that some individuals expressed uncertainty about PrEP uptake; to our knowledge there has been no prior systematic effort to examine key components of PrEP acceptability in this population, which is often needed when implementing new healthcare interventions (46). In this regard, Biello et al. found that initiatives to educate prospective PrEP users about the medications and about individual HIV risk would provide an essential mechanism to support PrEP, particularly in areas in which there is little existing knowledge about PrEP (23). Importantly, Furukawa et al. noted that adapting non-stigmatizing communication material that is appropriate for the population at risk of HIV is crucial for its acceptance (49). Given the dearth of PrEP educational materials currently designed for PWID, this would appear to be critically important to pursue. Educational efforts to create awareness and recalibrate perceptions of risk, incorporating specific discussion of high-risk situations may help to overcome uncertainty in gauging personal risk and managing prevention.

Our findings clearly demonstrated pervasive stigma and social exclusion that impacts rural PWID, in some cases undermining the traditionally close social bonds in rural communities, and effectively removing PWID from the protections of community membership. In particular, participants noted extensive enacted stigma from members of the law enforcement community involving policing practices that targeted them for enhanced surveillance. This was especially common among males that we interviewed, who tended to express enhanced concern about law enforcement scrutiny when compared to their female counterparts, tied to their lived histories of incarceration. With respect to law enforcement in particular, robust research has documented the harmful associations of harsh policing practices and increased risk for HIV among PWID (47, 48); this appears to be an especially salient concern in small rural communities where individuals are both well-known to and readily identifiable by police. Consistent with other recent research (37, 50, 51), participants of all ages and genders reported experiences of enacted stigma and dignity attacks in multiple settings, which they associated with reduced engagement with healthcare and treatment. As noted by Walters et al. (52), pervasive social exclusion is likely to play a large role in inequitable access to PrEP. Our findings resonate with recent research that has identified *exclusion from safety* as a driver of risk in marginalized populations (53) resulting from overt surveillance and discriminatory practices.

Participants concerns about scrutiny and stigma based on their background experiences of social exclusion had a strong connection to their expressed HIV prevention attitudes. We found that stigma related to HIV arose as a direct concern for PrEP uptake, with several participants reflecting that privacy issues would impact their preferences for education, prescribing and monitoring of PrEP. Most expressed a preference for one-on-one, and in-person PrEP education for privacy reasons and were enthusiastic about PrEP integration in the SSPs. These findings align with Allen et al. (21), who demonstrated that integration of PrEP services into venues that PWID already access would serve as a major support for communities with little knowledge of or access to PrEP. Among our sample, SSPs were widely considered safe spaces and trusted locations to receive services, and individuals expressed comfort and security attending these programs. Integration of PrEP care into existing SSPs would represent a structural expansion of the current service model at point of care, essentially creating an enabling environment for HIV prevention (35) and providing a seamless pathway for entry to PrEP care (54).

Strengthening and expanding the care system in rural SSPs to support PrEP services will require attention to adequately resourcing these locations. As observed in the present study, many rural PWID experience resource constraints, or limited physical capital. Conceptualized by White and Cloud (55), physical capital consists of the resources available to fulfill a person's basic needs, including healthcare, financial resources, clothing, food, safe shelter, and transportation. In this sample, physical capital barriers to PrEP uptake were common, but were mitigated to some extent by the presence of family housing and nearly universal health insurance coverage due to Kentucky's Medicaid expansion. Nevertheless, economic resources were extremely scarce, which deeply impacted access to reliable transportation and ability to pay for consistent phone or internet service, which allows people to connect with needed healthcare in an ongoing way.

Among the most notable supports for PrEP care were universal health insurance coverage, consistent pharmacy access, and histories of successful health management for other conditions. Kentucky's position as a southern Medicaid expansion state has afforded greater insurance and prescription benefit coverage among PWID, which will be critical for expansion of PrEP services and effectively reduced SSP clients' concerns about costs of PrEP medication. Removal of this structural barrier has contributed heavily to clients' experience of expanded access to healthcare and treatment services; unfortunately, we documented that many care episodes were adversely impacted by stigma and noted that clients reported improved engagement in care when providers were known, trusted, and empathetic, which supported open, non-judgmental communication. This finding is consistent with prior research demonstrating the importance of a robust therapeutic alliance for optimal HIV care planning (56) and fostering engagement and patient agency and activation in the care process (57). In this regard, recent research on HCV treatment and cure among PWID has documented important non-clinical impacts of treatment for health and wellbeing, including increased agency, confidence, and empowerment [(58)], which resonates with our finding that episodes of successful health management appear poised to support increased readiness for PrEP uptake.

## Limitations

This study is limited by a number of factors, including that it is heavily context-dependent, providing a snapshot of PWID's lived experiences in rural Kentucky communities that are both economically distressed and in the midst of a longstanding substance use epidemic, and operating in a policy environment that may be unique when comparted with communities in other locations. Second, given that interview participants were recruited from SSPs, they are not necessarily inclusive of all PWID in the targeted communities; this group may differ in important ways from PWID who do not utilize community harm reduction services. Finally, these narrative accounts are self-reports, which may be impacted by social desirability, self-presentation, and recall biases to an unknown extent. Assurances of confidentiality and the use of experienced neutral interviewers were employed to mitigate these potential deficiencies in self-report data.

## Conclusion

Drawing on the critical perspectives of people with lived experience, our findings provide important and actionable information on individual and environmental barriers and facilitators of PrEP uptake among rural PWID at high risk for HIV infection. These data will drive the adaptation and implementation of a client-centered approach to integrated PrEP care within rurally located SSP settings to address unmet needs for PrEP care.

## Data Availability Statement

The datasets presented in this article are not readily available because the data are in-depth qualitative interview transcripts, which due to their detail, are potentially identifiable. Requests to access the datasets should be directed to hilary.surratt@uky.edu.

## Ethics Statement

The studies involving human participants were reviewed and approved by University of Kentucky Medical Institutional Review Board. The patients/participants provided their written informed consent to participate in this study.

## Author Contributions

HS designed the study, obtained funding, led the data analysis, and wrote the first draft of the manuscript. HY assisted with data coding and preparation of the manuscript. AA, EG, EN, and TW assisted with data coding, reviewed, and revised the manuscript. All authors have reviewed and approved the final manuscript.

## Funding

This study was funded by NIH Grant Number R34DA053140.

## Author Disclaimer

The content is solely the responsibility of the authors and does not necessarily represent the official views of the National Institutes of Health.

## Conflict of Interest

The authors declare that the research was conducted in the absence of any commercial or financial relationships that could be construed as a potential conflict of interest.

## Publisher's Note

All claims expressed in this article are solely those of the authors and do not necessarily represent those of their affiliated organizations, or those of the publisher, the editors and the reviewers. Any product that may be evaluated in this article, or claim that may be made by its manufacturer, is not guaranteed or endorsed by the publisher.
